# A Dissemination Strategy to Identify Communities Ready to Implement a Pediatric Weight Management Intervention in Medically Underserved Areas

**DOI:** 10.5888/pcd18.200248

**Published:** 2021-02-11

**Authors:** Caitlin A. Golden, Jennie L. Hill, Kate A. Heelan, R. Todd Bartee, Bryce M. Abbey, Ali Malmkar, Paul A. Estabrooks

**Affiliations:** 1 University of Nebraska Medical Center, Omaha, Nebraska; 2 University of Nebraska at Kearney, Kearney, Nebraska

## Abstract

**Purpose and Objectives:**

We developed a competitive application process to test the feasibility of a fund and contract dissemination strategy to identify and engage communities that demonstrated the necessary resources and motivation to adopt, implement, and sustain a pediatric weight management intervention, Building Healthy Families, in rural and micropolitan (<50,000 residents) communities in Nebraska.

**Intervention Approach:**

From April through December 2019, a community advisory board with representation from rural and micropolitan clinical, public health, education, and recreational organizations collaboratively developed a request for applications, as a fund and contract dissemination strategy, to encourage community adoption of Building Healthy Families.

**Evaluation Methods:**

Quantitative assessments included determining the distribution of requests for applications, evaluating organizational readiness to change assessment (ORCA) ratings (on a scale of 1 to 5, from strongly disagree to strongly agree that the organization is ready to change), and reviewing community advisory board member ratings of applications. We gathered qualitative data from community narratives provided in response to the request for applications and community advisory board reviews of the applications.

**Results:**

The request for applications was distributed to all 93 counties in Nebraska. Of the 8 communities that submitted a letter of intent, 7 submitted a community narrative. Across the 8 communities, 31 ORCAs were completed by the organizational decision makers (n = 15) and staff members (n = 16) who would be responsible for screening, recruiting, or implementing the intervention. Overall mean ORCA scores varied by ratings of evidence (4.1–4.6), context (4.2–4.9), and facilitation (4.3–4.8), indicating a high degree of readiness. Community advisory board ratings of applications ranged from 2.3 to 3.4 of 4 points. Qualitative data indicated that lower community narrative scores were primarily caused by weak implementation and sustainability plans.

**Implications for Public Health:**

Findings provide guidance for translating pediatric weight management programs in medically underserved geographic areas by maximizing the probability of successful adoption and implementation through a fund and contract dissemination strategy.

**Summary**

**What is already known on this topic?**

Geographical disparities exist in the prevalence and treatment of childhood obesity. Expanding the availability of effective programming and expertise is imperative to translate research to practice in medically underserved areas.

**What is added by this report?**

This study describes a dissemination strategy and a systematic approach to identify communities that are ready and have demonstrated the capacity to disseminate a pediatric weight management intervention.

**What are the implications for public health practice?**

Findings provide guidance for translating pediatric weight management programs in medically underserved geographic areas by maximizing the probability of successful adoption and implementation through a community application process.

## Introduction

Childhood obesity prevalence is elevated across the United States and continues to be a pressing public health concern despite substantial prevention and treatment efforts (1). Disparities persist in obesity prevalence among children who have lower socioeconomic status and are in racial and ethnic minority groups (2–4). Furthermore, children residing in rural areas have 26% greater odds of obesity than their urban counterparts (3), and the most recent childhood obesity treatment recommendations do not address barriers for those living in medically underserved geographic areas (5).

Pediatric weight management interventions (PWMIs) are shown to reduce child weight (5–7). Efficacious PWMIs are family-based; they engage both the parent and child together and separately (8) through improved dietary intake, increased physical activity, and delivery of behavioral strategies through a multidisciplinary team (9,10). Most efficacious PWMIs were developed and implemented in large cities and urban areas and delivered through multidisciplinary teams in a hospital or medical center. For families living in micropolitan (ie, cities with populations <50,000) and rural areas, community resources and the teams needed to implement PWMIs are often not available (2).

The few PWMIs tested in efficacy trials in rural communities resulted in significant reductions of BMI *z* scores or percentile rankings (11). However, evidence is limited on the degree to which these or other efficacious PWMIs can be translated to, and are feasible in, other medically underserved geographic areas without adapting the interventions to the level of resources available and accessibility of multidisciplinary teams to deliver them (2,5,12). Currently, options are limited for childhood obesity treatment programs in Nebraska. These programs and other nearby programs outside the state are in hospital-associated metropolitan settings and require families to travel great distances. For example, a family living in the center of Nebraska, a rural area, who searches for a childhood obesity treatment program would find one in Omaha (a distance of 165 miles), Kansas City, Missouri (a distance of 265 miles), and Denver, Colorado (a distance of 310 miles). Building Healthy Families (BHF), an adapted evidence-based, family-based PWMI (10), was developed and implemented in a midwestern micropolitan city to provide a treatment option in medically underserved geographic areas for families with children who are obese. The magnitude of change in weight status of children participating in BHF was similar to the magnitude seen in efficacy trials (BMI *z* score reduction of ≥0.25) (10). Expanding the availability of effective programming and expertise and identifying the demand for PWMIs is imperative to translate research to practice in rural and micropolitan areas.

Numerous strategies have been developed to support dissemination and implementation of evidence-based interventions (EBIs) (13,14). Dissemination strategies that focus on organizational and community adoption and that include system-level and provider-level incentives have been used to help facilitate initial uptake (15). One dissemination strategy that holds promise is the use of organizational incentives to increase adoption of EBIs (16). Organizational incentives can take numerous forms (eg, payment schemes, ability to bill for the innovation or provide as a fee for service) (13). However, few have been applied to PWMIs or outside metropolitan clinical or health care settings (14,17).

A method for organizational incentives that may be practical in underresourced areas is a fund and contract dissemination strategy (13). This method includes a competitive funding announcement and a modest budget to identify and engage communities that have demonstrated the necessary resources and motivation to adopt, implement, and sustain evidence-based practices (13). This strategy allows limited resources to be allocated to communities or organizations most ready to act successfully on those resources to increase the likelihood of PWMI adoption, implementation, and sustainability. A fund and contract strategy is predicated on a pull, rather than a push, approach to increase community uptake of an evidence-based PWMI. Pull approaches aim to identify delivery systems that prioritize a given issue and are motivated and ready to implement an EBI (18). Push approaches bring EBIs to systems that have a need for a program but may not prioritize the issue addressed by the EBI. Push approaches may require substantial system changes in communities that are not ready for change and can inhibit successful adoption, implementation, and sustainability of EBIs (18).

In addition to needing to determine the utility of dissemination strategies to successfully engage organizations and communities in adopting an evidence-based approach, the underlying mechanisms that facilitate an adoption decision need to be explored (19). Organizational readiness for change has been theorized as an important precursor that influences successful adoption of evidence-based approaches in the Promoting Action on Research in Health Services (PARIHS) model (20,21). Studies using the organizational readiness to change assessment (ORCA) in clinical settings demonstrated that evidence, context, and facilitation predict use of EBIs (21). Organizations with high scores across these 3 constructs are more likely to be successful in adopting, implementing, and sustaining an EBI (22).

## Purpose and Objectives

The purpose of this study was to test the feasibility of a fund and contract dissemination strategy for a PWMI in identifying and engaging communities to adopt BHF in rural and micropolitan areas of Nebraska. Feasibility was operationalized as the ability of the strategy to engage 4 to 8 geographically dispersed communities to commit to delivering BHF. A secondary purpose was to describe the organizational readiness for change of communities — based on ORCA scores and BHF community advisory board (CAB) assessments of communities that responded to the request for applications (RFA). We hypothesized that 1) the dissemination strategy would lead to the identification of a broad cross-section of communities and community organizations interested in PWMI delivery, 2) the inclusion of requirements for formal implementation commitments and engagement across community organizations would reduce the number of communities that would transition from a letter of intent to a full application call for proposals, and 3) communities that submit a full application for proposals would report high scores.

### Intervention approach

This study was part of a larger hybrid type III effectiveness–implementation pilot study to test the adoption, implementation, and sustainability of BHF. A secondary aim of the larger trial is to determine the effectiveness of BHF in reducing child weight. Approval for the study was provided by the University of Nebraska at Kearney Institutional Review Board. The larger trial focuses on a collaborative approach to package all BHF program implementation and training resources necessary to support adoption and implementation of the program in new micropolitan and surrounding rural communities. Additionally, the larger trial includes implementing the packaged program in communities with and without participation in a learning collaborative. The phase of the project described in this study examined the utility of a dissemination strategy intended to identify communities that were motivated and ready to adopt a new PWMI. Specifically, we tested a fund and contract strategy that included a competitive process for organizations that serve low-income families in medically underserved communities to apply for access to the BHF program and resources.

To increase the likelihood of successful adoption of BHF, we used a systems-based approach to incorporate multiple sectors and vertical structures (ie, within organizations) within each community and community-based organization (23). This approach allows for engagement of community partners to increase referrals among children and families, identify the available community resources to implement BHF, and determine the likelihood of BHF aligning with community values and long-term sustainability (24,25).

## Evaluation Methods

We used ORCA scores to quantitatively assess community readiness. BHF-CAB members also provided a quantitative rating of community narratives. Finally, qualitative data were gathered from community narratives and BHF-CAB member reviews of the narratives. This phase of the project was initiated in April 2019, and community award announcements were made in December 2019.

### Setting and participants

Communities were eligible to participate in this study if they were located in micropolitan (population of at least 10,000 but fewer than 50,000) and surrounding rural communities (population of at least 50,000) outside the 2 largest metropolitan areas in Nebraska (Lincoln, population ~334,590; Omaha, population ~942,198). Ninety of 93 counties in Nebraska were eligible to submit a letter of intent and apply for funding; 3 counties were not eligible because they were metropolitan. Any community organization in an eligible county that could demonstrate local need and potential infrastructure to recruit families and implement BHF was eligible to apply.

### Procedures

Community members invited to serve on the BHF-CAB included representatives from regional public health networks, community and health care organizations, people with experience implementing or participating in BHF, and representatives from an interdisciplinary research team. The overarching goal was to develop a CAB with strong cross-system representation for rural Nebraska. This 19-member CAB was developed as part of the larger trial, with the goal to contribute to all aspects of this research. A 3-phase approach was used to determine regional demand, motivation, and commitment to adopt, implement, and sustain BHF. Phase 1 emphasized a horizontal systems (ie, between organizations) approach to identify and build on partnerships with strong working relationships across community organizations.

The BHF-CAB members collaboratively developed an RFA that included the submission of a letter of intent and a full community narrative. A list of first contacts was strategically developed for RFA distribution through email across the BHF-CAB member networks throughout Nebraska. Recipients of the email were asked to forward the information to their contacts who expressed interest in providing a PWMI in their community. Those who forwarded the RFA were asked to report back to the BHF-CAB the number of contacts and organizations that received the RFA information. We tracked the number of organizations that received the RFA directly from BHF-CAB members. However, dissemination was likely broader than reported by BHF-CAB members, because organizations that received the RFA were also encouraged to send it to other groups. As a result, we used the total number of eligible counties (n = 90) as our denominator for dissemination and the number of counties with organizations that received the RFA as the numerator. In addition to email distribution, we created a website to promote the pilot study and provide information on the 2-step RFA process, a timeline, and frequently asked questions for organizations interested in applying for funding.

Phase 2 was designed to assess local demand for the packaged PWMI and to identify the potential determinants of adoption of BHF. In this phase, we used a letter of intent procedure to gather information on the demand for BHF followed by a full application procedure with more rigorous requirements for participation. Descriptions of BHF and the potential relative advantage of a packaged approach were shared with communities through the RFA materials. The overall goal of this phase was to promote a systems-based approach by requiring communities to document multisectoral partnerships and vertical representation from partners that would be involved in screening and recruiting families and implementing and sustaining BHF. Each participating organization submitted a letter of intent and was required to have a minimum of 2 members from each organization complete a modified ORCA: 1 person with organizational decision-making authority and 1 person who would be responsible for implementing BHF.

The ORCA is an instrument designed to measure the evidence, context, and facilitation constructs of the PARIHS framework, which are theorized to predict implementation outcomes (21). We used a modified 50-item version of the ORCA to assess community readiness to implement BHF (Table 1). The evidence scale assessed respondent ratings of the strength and extent of evidence for PWMIs across 3 subscales: research evidence, clinical experience, and patient preferences (26). Modifications to the scale included framing clinical experience and patient preferences as community experience and community member preferences. The context scale consisted of 6 subscales assessing organizational culture, leadership, measurement, resources, and readiness to change among opinion leaders (27). Lastly, the facilitation scale addressed the capacity for internal facilitation and consisted of 4 subscales assessing leadership characteristics and roles, project champion characteristics, and implementation team roles (28). All items were assessed on a 5-point Likert scale, from strongly disagree to strongly agree.

**Table 1:** Organizational Readiness to Change Assessment (ORCA)^a^ Items Used to Assess Communities in Nebraska Interested in Adopting and Implementing Building Healthy Families, a Pediatric Weight Management Intervention, 2019

Scale and Subscale	Item
**Evidence**	
Research	Implementing Building Healthy Families in my community:
	• Is supported by strong scientific evidence in communities like mine.
	• Is supported by strong scientific evidence from other communities that may not be like mine.
	• Should be effective, based on strong scientific evidence from my community, or other communities like mine.
Community experience	The decision to implement Building Healthy Families:
	• Is supported by my experience with my community and its residents.
	• Is supported by similar experience with residents in other communities.
	• Matches the opinions of experts in my community.
Community preference	The decision to implement Building Healthy Families:
	• Would be/has been well-received by community members in a pilot study.
	• Is consistent with programs that have been accepted by community residents.
	• Takes into consideration the needs and preferences of my community.
	• Appears to have more advantages than disadvantages for my community.
**Context**	
Leadership culture	Senior leadership/clinical management in your organization:
	• Reward innovation and creativity to improve community health.
	• Solicit opinions of staff regarding decisions about contributing to community health.
	• Seek ways to improve community health and increase community resident participation in programs.
Staff culture	Staff members in your organization:
	• Have a sense of personal responsibility for improving community health.
	• Cooperate to maintain and improve effectiveness of community health programs.
	• Are willing to innovate and/or experiment to improve how things are done.
	• Are receptive to change in community offerings and processes.
Leadership	Senior leadership/management in your organization:
	• Provide effective management to improve community health.
	• Clearly define areas of responsibility and authority for managers and staff.
	• Promote team building to solve community program problems.
	• Promote communication among organizational services and units.
Measurement	Senior leadership and management in your organization:
	• Provide staff with information on their performance measures and guidelines.
	• Establish clear goals for processes and outcomes.
	• Provide staff members with feedback/data on effects of their decisions.
	• Hold staff members accountable for achieving results.
Readiness for change	Opinion leaders (people who influence the opinions, attitudes, beliefs, motivations, and behaviors of others) in your organization:
	• Believe that how you currently address childhood obesity can be improved.
	• Encourage and support changes in your approach to childhood obesity.
	• Are willing to try new community programs.
	• Work cooperatively with senior leadership/management to make appropriate changes.
Resources	In general, in my organization, when there is agreement that change needs to happen:
	• We have the necessary support in terms of budget or financial resources.
	• We have the necessary training support.
	• We have the necessary facilities support.
	• We have the necessary staffing support.
**Facilitation**	
Leader characteristics	Senior leadership/management will:
	• Propose a project that is appropriate and feasible.
	• Provide clear goals for improving community health.
	• Establish a project schedule and deliverables.
	• Designate an organizational champion(s) for the project.
Project champion characteristics	The childhood obesity treatment project champion (your community lead):
	• Accepts responsibility for the success of this project.
	• Has the authority to carry out the implementation.
	• Is considered an organizational opinion leader.
	• Works well with the intervention team and partners.
Leadership implementation roles	Senior Leadership/management/staff opinion leaders:
	• Agree on the goals for this program.
	• Will be informed and involved in the program planning and implementation.
	• Agree on adequate resources to implement the program.
	• Set a high priority on the success of the program.
Implementation team roles	The potential implementation team members:
	• Share responsibility for the success of this project.
	• Have clearly defined roles and responsibilities.
	• Have release time or can accomplish intervention tasks within their regular workload.
	• Have staff support and other resources required for the project.

a The ORCA is an instrument designed to measure the evidence, context, and facilitation constructs of the Promoting Action on Research in Health Services (PARIHS) model (20,21), which are theorized to predict implementation outcomes. We used a modified 50-item version of the ORCA to assess community readiness to implement Building Healthy Families, a pediatric weight management intervention.

Participants responded to questions about their perceptions of the strength of evidence for BHF and the community context and facilitation that would support implementing the PWMI. We calculated baseline means for overall ORCA scales and subscale scores for evidence, context, and facilitation for each community. Community-perceived readiness from the ORCA was operationalized as “ready” if the mean scale and subscale scores were greater than 4.0, “somewhat ready” if the mean scores were greater than 3.0 but less than 4.0, and “not ready” if the mean scores were 3.0 or less. The ORCA responses were used to characterize readiness, but to reduce the likelihood of social desirability biases, the communities were informed that the responses would not be considered as part of the evaluation of the letters of intent. All respondents completed the survey in a de-identified, online format so information was not shared among applicants.

One week after the letters of intent and ORCAs were submitted, an informational webinar further detailing BHF and the community requirements for participating in the implementation pilot study was provided for communities that submitted a letter of intent. If communities were unable to attend the webinar, they were encouraged to reach out to the program coordinator or refer to the frequently asked questions section of the website. After the webinar, communities that submitted a letter of intent were given a month to complete and submit a full application and community narrative.

Phase 3 aimed to identify, by using the community narratives, communities that were ready to pilot test BHF. Communities that submitted narratives were asked to demonstrate 1) the local priority or need for a PWMI, 2) their ability to develop recruitment methods, 3) their ability to implement BHF, and 4) their plan for sustainability in their community. Each community was also required to 1) provide documentation of their service to low-income families, 2) identify their multisectoral partnerships, 3) agree to implement 2 or 3 cohorts of BHF, 4) use pragmatic evaluation strategies throughout the implementation pilot study, and, if selected 5) participate in a learning collaborative. Formal commitment (a written memorandum of understanding) from each community was also required from those that submitted the community narrative and were selected to participate.

The BHF-CAB members evaluated the community narrative submissions and scored the community applications. The average scores were calculated for each community application; ORCA responses were not provided or used as part of evaluation. Each reviewer was provided an evaluation form with scoring criteria for each section of the application. Ratings were made on a scale of 0 to 4, with 0 indicating a very weak section and 4 indicating a very strong section. Additionally, qualitative feedback was requested from each reviewer for key factors that informed the ratings. The community members of the BHF-CAB were each assigned a minimum of 2 and up to 4 narratives to review and score, to reduce community burden or conflicts of interest. BHF-CAB research team members evaluated all of the narrative submissions. An average was calculated from the BHF-CAB community member evaluations and the research team member evaluations for an overall score and application rank. Community readiness from the narrative applications were operationalized as “ready” if the mean scores were 3.0 or greater, “somewhat ready” if the mean scores were 2.0 to less than 3.0, and “not ready” (ie, the application had weaknesses that could negatively affect implementation) if the community did not submit a narrative or the mean scores were less than 2.0.

## Results

### Phase 1: CAB development and RFA distribution

**Figure 1:**
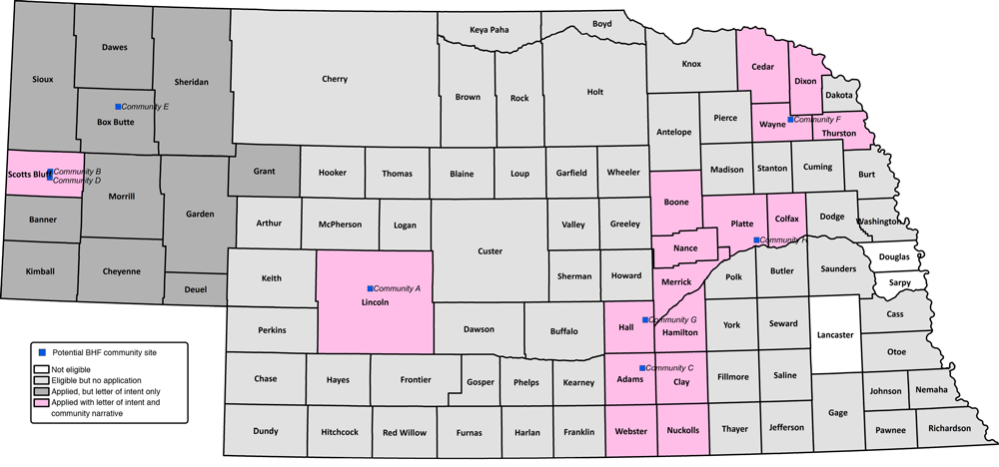
Counties deemed eligible (n = 90) and response to the request for application among counties interested in adopting and implementing Building Healthy Families, a pediatric weight management intervention, Nebraska, 2019.

The average number of organizations or people that received notification about the RFA from each BHF-CAB member was 6, with a range of 2 to 9. Organizations and people that received notification of the RFA included cooperative extension personnel (n = 39), departments of public health (n = 15), regional hospitals (n = 11), community recreation organizations (n = 10), federally qualified health centers (n = 8), nonprofit organizations (n = 8), and public school districts (n = 5). Based on the locations of organizations or people that received notification, the BHF-CAB members distributed the RFA statewide (n = 93 counties) (Figure 1).

### Phase 2: Determining local demand for a packaged PWMI program and training resources

In the first step of our 2-step request for applications process, 8 letters of intent were received from communities interested in adopting and implementing BHF (Table 2). Across those communities, 31 ORCAs were completed, 15 by organizational decision makers and 16 by staff members who would be responsible for screening, recruiting, or implementing the PWMI. The communities that submitted letters of intent represented 28 (31%) of 90 eligible counties. No 2 communities had the same mix of organizational partnerships.

**Table 2:** Characteristics of the 8 Communities in Nebraska That Submitted a Letter of Intent Expressing Interest in Adopting and Implementing Building Healthy Families, a Pediatric Weight Management Intervention, 2019

Characteristic	Community							
	A	B	C	D	E	F	G	H
**Population**	35,185	35,989	45,453	35,989	84,801	30,906	78,620	53,105
**No. of counties**	1	1	4	1	12	4	3	4
**Race/ethnicity, %**								
White (non-Hispanic)	87.2	71.6	86.9	71.6	79.2	77.2	71.2	73.9
Hispanic	9.1	24.2	9.5	24.2	15.4	6.8	23.7	22.6
Black or African American	1.3	1.0	1.0	1.0	1.0	0.9	3.1	2.1
American Indian/Alaska Native	1.1	3.5	1.2	3.5	3.5	14.5	1.8	1.7
≥2 Races	1.6	1.6	1.3	1.6	2.0	1.6	1.5	1.2
Other	1.0	1.0	1.2	1.0	1.0	0.5	1.7	1.2
**Median community income, $**	55,875	50,157	46,888	50,157	45,761	57,122	54,742	55,191
**Institutional role of person who completed the ORCA**								
Decision maker	1	1	1	1	1	2	1	1
Program implementer	3	3	4	2	1	3	5	1
**Type of organization submitting letter of intent**	Hospital, recreation	Hospital	Education, health department, hospital, recreation	Community health center	Health department	Health department, hospital	Education, health center, health department	Health department
**Team member position**	Nurse coordinator, recreation director and employee, wellness educator	Chief operating officer, medical director, physician, recruitment coordinator	Chief executive officer, executive director, extension educator, program coordinator, wellness manager	Advanced practice registered nurse, clinic director, registered dietitian	Community health director, deputy director	Chief executive officer, chief nursing officer, health director, program coordinator	Accreditation coordinator, associate superintendent, chief executive officer, health director, medical director, outreach liaison	Chief public health officer, WIC nutritionist

Abbreviation: WIC, Special Supplemental Nutrition Program for Women, Infants, and Children.

**Figure 2:**
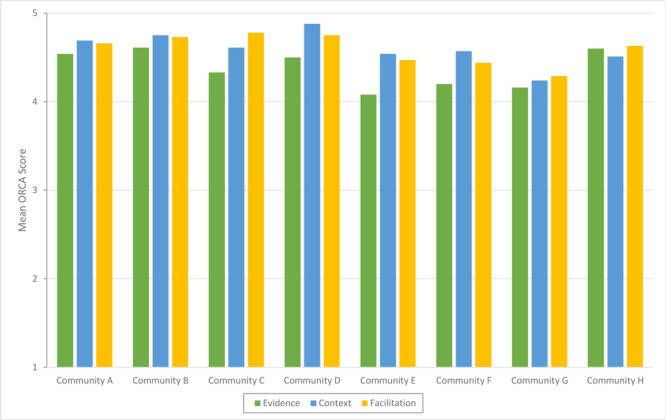
Organizational readiness to change assessment (ORCA) scores in the 8 communities that submitted a letter of intent expressing interest in adopting and implementing Building Healthy Families, a pediatric weight management intervention, Nebraska, 2019. Readiness was operationalized as “ready” if the mean scale and subscale scores were greater than 4.0, “somewhat ready” if the mean scores were greater than 3.0 but less than 4.0, and “not ready” if the mean scores were 3.0 or less.

We found variability in community ratings of readiness based on the ORCA completion. However, the overall perceptions of community team members indicated that they were ready to implement BHF (Figure 2). Overall readiness mean (SD) scores for communities, by construct (of a possible 5 points) were highest for context (4.6 [0.5]) and facilitation (4.6 [0.5]) followed by evidence (4.4 [0.4]). The largest variability in perceived readiness for communities was the subscale general resources (4.3 [0.6]). This subscale assessed a community’s perceived availability of resources to implement BHF (staff incentives, equipment and materials, participant awareness/need, instructor buy-in, intervention team, and evaluation protocols).

**Table:** 

Community	Evidence	Context	Facilitation
Community A	4.5	4.7	4.7
Community B	4.6	4.8	4.7
Community C	4.3	4.6	4.8
Community D	4.5	4.9	4.8
Community E	4.1	4.5	4.5
Community F	4.2	4.6	4.4
Community G	4.2	4.2	4.3
Community H	4.6	4.5	4.6

**Figure 3:**
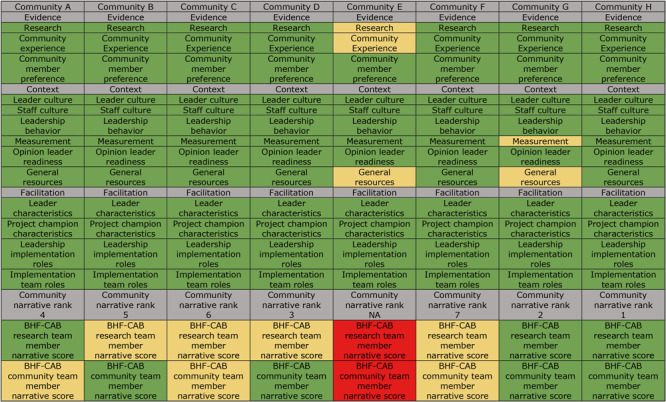
Community readiness to implement Building Healthy Families (BHF), a pediatric weight management intervention, Nebraska, 2019. Readiness was operationalized as “ready” (in green) if the mean scale and subscale scores were greater than 4.0, “somewhat ready” (in yellow) if the mean scores were greater than 3.0 but less than 4.0, and “not ready” (in red) if the mean scores were 3.0 or less. Abbreviations: CAB, community advisory board; NA, not applicable.

Based on the ORCA subscale scores, 6 communities (all but Community E and Community G) were rated as ready for implementation. One community that submitted a letter of intent, community E, decided to discontinue its application process after the community webinar. It was rated as “somewhat ready” for evidence subscales for research evidence and community experience as well as for general resources. Similarly, Community G was rated as “somewhat ready” for context subscales of general resources and measurement (Figure 3).

**Table:** 

Community A	Community B	Community C	Community D	Community E	Community F	Community G	Community H
Evidence	Evidence	Evidence	Evidence	Evidence	Evidence	Evidence	Evidence
Research (ready)	Research (ready)	Research (ready)	Research (ready)	Research (somewhat ready)	Research (ready)	Research (ready)	Research (ready)
Community experience (ready)	Community experience (ready)	Community experience (ready)	Community experience (ready)	Community experience (somewhat ready)	Community experience (ready)	Community experience (ready)	Community experience (ready)
Community member preference (ready)	Community member preference (ready)	Community member preference (ready)	Community member preference (ready)	Community member preference (ready)	Community member preference (ready)	Community member preference (ready)	Community member preference (ready)
Context	Context	Context	Context	Context	Context	Context	Context
Leader culture (ready)	Leader culture (ready)	Leader culture (ready)	Leader culture (ready)	Leader culture (ready)	Leader culture (ready)	Leader culture (ready)	Leader culture (ready)
Staff culture (ready)	Staff culture (ready)	Staff culture (ready)	Staff culture (ready)	Staff culture (ready)	Staff culture (ready)	Staff culture (ready)	Staff culture (ready)
Leadership behavior (ready)	Leadership behavior (ready)	Leadership behavior (ready)	Leadership behavior (ready)	Leadership behavior (ready)	Leadership behavior (ready)	Leadership behavior (ready)	Leadership behavior
Measurement (ready)	Measurement (ready)	Measurement (ready)	Measurement (ready)	Measurement (ready)	Measurement (ready)	Measurement (somewhat ready)	Measurement (ready)
Opinion leader readiness (ready)	Opinion leader readiness (ready)	Opinion leader readiness (ready)	Opinion leader readiness (ready)	Opinion leader readiness (ready)	Opinion leader readiness (ready)	Opinion leader readiness (ready)	Opinion leader readiness (ready)
General resources (ready)	General resources (ready)	General resources (ready)	General resources (ready)	General resources (somewhat ready)	General resources (ready)	General resources (somewhat ready)	General resources (ready)
Facilitation	Facilitation	Facilitation	Facilitation	Facilitation	Facilitation	Facilitation	Facilitation
Leader characteristics (ready)	Leader characteristics (ready)	Leader characteristic (ready)s	Leader characteristics (ready)	Leader characteristics (ready)	Leader characteristics (ready)	Leader characteristics (ready)	Leader characteristics (ready)
Project champion characteristics (ready)	Project champion characteristics (ready)	Project champion characteristics (ready)	Project champion characteristics (ready)	Project champion characteristics (ready)	Project champion characteristics (ready)	Project champion characteristics (ready)	Project champion characteristics (ready)
Leadership implementation roles (ready)	Leadership implementation roles (ready)	Leadership implementation roles (ready)	Leadership implementation roles (ready)	Leadership implementation roles (ready)	Leadership implementation roles (ready)	Leadership implementation roles (ready)	Leadership implementation roles (ready)
Implementation team roles (ready)	Implementation team roles (ready)	Implementation team roles (ready)	Implementation team roles (ready)	Implementation team roles (ready)	Implementation team roles (ready)	Implementation team roles (ready)	Implementation team roles (ready)
Community narrative rank 4	Community narrative rank 5	Community narrative rank 6	Community narrative rank 3	Community narrative rank NA	Community narrative rank 7	Community narrative rank 2	Community narrative rank 1
BHF-CAB research team member narrative score (ready)	BHF-CAB research team member narrative score (somewhat ready)	BHF-CAB research team member narrative score (somewhat ready)	BHF-CAB research team member narrative score (somewhat ready)	BHF-CAB research team member narrative score (not ready)	BHF-CAB research team member narrative score (somewhat ready)	BHF-CAB research team member narrative score (ready)	BHF-CAB research team member narrative score (ready)
BHF-CAB community team member narrative score (somewhat ready)	BHF-CAB community team member narrative score (ready)	BHF-CAB community team member narrative score (somewhat ready)	BHF-CAB community team member narrative score (ready)	BHF-CAB community team member narrative score (not ready)	BHF-CAB community team member narrative score (somewhat ready)	BHF-CAB community team member narrative score (ready)	BHF-CAB community team member narrative score (ready)

### Phase 3: Identify communities ready to pilot test the utility of a packaged PWMI and training materials through a community narrative application

In the second step of our 2-step process, 7 communities that provide service across 17 counties submitted complete applications. Average overall BHF-CAB scores for communities ranged from 2.3 to 3.4 of a possible 4 points. We rank ordered communities by their overall score for demonstrating the need in their community and plans for recruitment, implementation, and sustainability (Figure 3). On average, readiness scores given by BHF-CAB community team members were higher than scores given by BHF-CAB research team members, although rank orderings of communities were nearly identical. Communities G and H scored the highest and were considered to be ready to implement BHF by both BHF-CAB research team members and BHF-CAB community members. BHF-CAB research team members and BHF-CAB community members differed in their scoring for communities A, B, and D. BHF-CAB community members gave community A lower scores than did BHF-CAB research team members and perceived them to be somewhat ready, whereas BHF-CAB research team members perceived them to be ready to implement BHF. Additionally, BHF-CAB community members gave higher scores to communities B and D than did BHF-CAB research team members and perceived them as ready, whereas BHF-CAB research team members perceived them as somewhat ready to implement BHF. Although the ORCA responses for community E indicated they were somewhat ready to implement BHF, they did not complete the application process by submitting a community narrative and were therefore considered not ready.

The qualitative data indicated that lower scores were primarily based on weak implementation and sustainability plans for BHF. Communities that had lower scores lacked details on recruitment and screening efforts and partnership development for implementation and sustainability. Communities that were ranked higher by BHF-CAB members demonstrated strong plans for implementation and established multisectoral partnerships for recruitment, screening, and delivery of BHF. Across all communities, applicants demonstrated areas of weaknesses in generating plans for sustainability. Those with positive sustainability ideas, such as integrating some components of BHF implementation into job descriptions, did not provide details beyond a simple description. Others simply stated they would pursue additional funding for sustainability. Future use of this dissemination strategy would be improved by providing more detailed questions on system strategies that would heighten the likelihood of sustainability.

## Implications for Public Health

The state of Nebraska has identified pediatric and adult obesity prevalence as a priority public health concern and aims to develop a statewide coordinated approach to reduce obesity in children, adults, and members of racial and ethnic minority groups (29). Expanding the availability of effective programming and expertise is imperative to translate research to practice in medically underserved geographic areas. Effective dissemination and implementation strategies are needed to identify and engage communities with the potential capacity to adopt, implement, and sustain BHF. The objective of this study was to test the feasibility of a fund and contract dissemination strategy for a PWMI in identifying and engaging communities to adopt BHF in rural and micropolitan areas of Nebraska. Based on the approach we used, we can make 3 primary generalizations from these data. First, a fund and contract strategy successfully generated a broad cross-section of communities and community organizations interested in delivering BHF and some potential capacity for implementation. Second, based on ORCA responses, community organizations appeared to have strong perceptions on the quality of the evidence on BHF, positive local contexts for implementation, and the likelihood of supportive facilitation infrastructure. Third, the community narrative phase of the application provided critical insights on the potential barriers and facilitators in communities that could affect implementation efforts.

Initial interest from communities coupled with funding for implementation activities, formal commitments, and implementation support has been shown to increase capacity for evidence-based approaches (30). Our observational approach extends these findings to demonstrate that this type of strategy can also be used to identify organizations ready to adopt a new EBI (31). We also found that a simple fund and contract strategy identified communities with good geographic representation across a broad rural region and set the stage to investigate whether this representation translates into PWMIs that reach families across the state.

If each identified community implements BHF, overall travel time to the new programs, even from the most distant areas, would require less time than is necessary to travel to the closest metropolitan areas with PWMI opportunities — Denver, Omaha, or Kansas City. In addition to addressing geographic barriers, a local PWMI would provide resources that might otherwise be limited in communities for families to receive obesity treatment and opportunities to engage in community-tailored physical activity and nutrition education together. Thus, a fund and contract strategy may be an effective tool for eliminating initial barriers to adopting a PWMI and can identify communities ready for implementation.

The positive ratings of evidence, context, and facilitation across communities is promising (32–34). Although we do not yet know if the identified communities will adopt and implement BHF with high quality, organizational readiness — defined as the interplay between ratings of evidence, context, and facilitation — has been identified as one of the strongest predictors of successful adoption and implementation of EBI using the PARIHS framework (20,35,36). Whether the fund and contract dissemination strategy facilitated organizational readiness and communities’ capacity to implement BHF is unclear. The strategy may have simply uncovered communities that potentially would have adopted a PWMI without this process or encouraged a more positive view of readiness with the excitement of engaging new partners in the respondent communities. As the project moves forward, additional assessment of organizational readiness and qualitative interviews with communities during the pre/post-adoption and pre/post-implementation stages is expected to provide information to determine whether a relationship exists between initial assessments and the likelihood of successful implementation (36,37).

The qualitative narrative process required each applicant to describe their readiness and capacity and to initiate and sustain partnership development with the purpose of addressing childhood obesity. Relative to the initial quantitative information provided by the candidates, the qualitative information provided correspondence for communities with high readiness ratings and gave potential causes for communities with low readiness ratings. Common areas of weakness for communities were due to limited data to identify low-income families with children who have obesity, insufficient plans for recruitment and partners for recruitment efforts, no established or pre-identified partners and defined personnel roles, and insufficient sustainability plans. This information provided a better understanding of community context and target areas for implementation strategies to further engage communities in their dissemination and implementation planning process to increase the likelihood of long-term sustainability (38).

Our descriptive study explored hypotheses of whether a fund and contract dissemination strategy can be used to identify a geographically dispersed set of communities with the potential to adopt, implement, and sustain an evidence-based PWMI. However, one limitation of a descriptive study is that it is intended to provide information to generate rather than test hypotheses. As such, although our findings aligned with the exploratory hypotheses, more rigorous experimental designs will be needed to test these hypotheses. The outcome of our study was the completion of the memorandum of understanding committing each community to implement BHF — and not actual adoption. Still, the value of our project lies in the novel use of a fund and contract strategy outside health care settings, the demonstration that this process can attract a geographically dispersed set of communities to commit to the implementation of BHF, and the provision of evidence that the fund and contract approach can facilitate cross-organizational and within-organization efforts to respond to a regional health priority.

Our study provides a systematic approach to identifying and engaging communities that are ready and able to disseminate BHF in their community to increase the likelihood of program adoption and implementation. The 3-phase process allowed community partnerships interested in disseminating BHF to identify communities with initial interest and, through a fund and contract dissemination strategy, narrow down the number of communities to those that are ready and have the apparent capacity to implement a PWMI in their community. Our novel approach to integrating a “pull strategy” through a competitive application process, including a letter of intent procedure followed by a full application narrative, allowed for the identification of 7 new communities that were ready to adopt and pilot the utility of a packaged PWMI and training resources.
